# Discovery of Porcine microRNAs in Multiple Tissues by a Solexa Deep Sequencing Approach

**DOI:** 10.1371/journal.pone.0016235

**Published:** 2011-01-25

**Authors:** Sheng-Song Xie, Xin-Yun Li, Teng Liu, Jian-Hua Cao, Qiang Zhong, Shu-Hong Zhao

**Affiliations:** Key Lab of Agricultural Animal Genetics, Breeding, and Reproduction of Ministry of Education & Key Laboratory of Swine Genetics and Breeding of Ministry of Agriculture, Huazhong Agricultural University, Wuhan, People's Republic of China; Istituto Dermopatico dell'Immacolata, Italy

## Abstract

The domestic pig (*Sus scrofa*) is an important economic animal for meat production and as a suitable model organism for comparative genomics and biomedical studies. In an effort to gain further identification of miRNAs in the pig, we have applied the Illumina Solexa sequencing technology to carry out an in-depth analysis of the miRNA transcriptome in a pool of equal amounts of RNA from 16 different porcine tissues. From this data set, we identified 437 conserved and 86 candidate novel miRNA/miRNA* in the pig, corresponding to 329 miRNA genes. Compared with all the reported porcine miRNAs, the result showed that 112 conserved and 61 candidate novel porcine miRNA were first reported in this study. Further analysis revealed extensive sequence variations (isomiRs) of porcine miRNAs, including terminal isomiRs at both the 5′ and 3′ ends and nucleotide variants. Read counts of individual porcine miRNA spanned from a few reads to approximately 405541 reads, confirming the different level of expression of porcine miRNAs. Subsequently, the tissue expression patterns of 8 miRNAs were characterized by Northern blotting. The results showed that miR-145, miR-423-5p, miR-320, miR-26a, and miR-191 are ubiquitously expressed in diverse tissues, while miR-92, miR-200a, and miR-375 were selectively enriched and expressed in special tissues. Meanwhile, the expression of 8 novel porcine-specific miRNAs was validated by stem-loop RT-PCR, and one of these was detected by Northern blotting. Using the porcine miRNA array designed according to our Solexa results, 123 miRNAs were detected expression in porcine liver tissues. A total of 58 miRNAs showed differential expression between the Tongcheng (a Chinese indigenous fatty breed) and Large White pig breeds (a lean type pig). Taken together, our results add new information to existing data on porcine miRNAs and should be useful for investigating the biological functions of miRNAs in pig and other species.

## Introduction

MicroRNAs (miRNAs) belong to a group of single-stranded noncoding RNAs that are 21–23 nucleotides (nt) in length [1]. The first characterized miRNA was *lin-4* in 1993, but miRNAs were not recognized as a distinct class of biological regulators with conserved functions until the early 2000s [2], [3], [4]. These molecules typically interact with target mRNAs through base pairing, predominantly between bases 2–8 (the “seed” region) at the 5′ end of the miRNA and complementary sites in the target 3′ untranslated regions (3′ UTR, “seed matches”) [5]. These interactions result in translational repression and/or deadenylation of target mRNA [6], [7]. In animals, a few miRNAs can regulate gene expression at the post-transcriptional level by encoding target protein mRNAs through perfect complementary base pairing that leads to mRNA degradation. A typical example is miR-196, which can direct the cleavage of HOXB8 mRNA in mouse embryos [8]. miRNA biogenesis occurs through a multistep process that involves the activities of two Ribonuclease IIIs called Drosha and Dicer [9], [10]. Many miRNAs are expressed in a tissue-specific and/or stage-specific manner [11]. A single miRNA can have one to several hundred target mRNAs, and it is estimated that 30% of all protein-coding genes in humans are regulated by miRNAs [5].

Increasing evidence suggests that miRNAs play important roles in cell differentiation, proliferation, growth, apoptosis, and immune response [12], [13], [14]. miRNAs regulate target mRNAs and act as rheostats that make fine-scale adjustments to protein output [15]. miRNAs are involved in the normal functioning of eukaryotic cells, and dysregulation of miRNAs is associated with disease [16]. Several miRNAs have links with certain types of cancer [17]. Extensive studies have indicated diverse patterns of miRNA regulation. Mature miRNAs target the 3′ UTR of genes by complementary base pairing [18]. Furthermore, mature miRNAs can alter gene expression by binding to the coding regions as well as to the 5′ UTR [19], [20]. The down-regulation of target mRNAs by miRNAs has been widely observed. Recent experiments also show that miRNAs can up-regulate target mRNAs in some cases [21]. However, the biological functions of most miRNAs and their precise regulatory mechanisms remain elusive. Therefore, much effort has been made to elucidate miRNA functions in recent years.

Pigs are essential organisms in the study of human health because their physiology is similar to that of humans, and they have comparable organ size and body mass. These animals are used as models for studying the consequences of infectious disease, testing drugs, practicing new surgical techniques, performing xenotransplants, and exploring lifestyle diseases such as cardiovascular disease and obesity [22], [23]. Despite increasing evidence for the diverse roles of miRNAs in biological processes, obtained through homology search or/and small RNA cDNA library cloning, the progress of miRNA studies in pigs has been slow [24], [25], [26], [27], [28], [29], [30], [31]. The number of porcine miRNAs explored has increased in recent times due to the application of new deep sequencing techniques such as those from 454 Life Sciences and Solexa, which are suitable for the sequencing of miRNAs. This has greatly accelerated porcine miRNA discovery [32], [33], [34]. However, identification of miRNAs in pigs has been limited to certain tissues such as the skeletal muscle, adipose, heart, liver, thymus, and intestinal tissues. We hypothesize that more miRNAs are present. In particular, pig-specific novel miRNAs may exist. To test our hypothesis, we constructed a small RNA cDNA library using pooled RNA from 16 porcine tissues, for example, heart, liver, spleen, lung, kidney, and skeletal muscle. These tissues were collected from the Meishan boar, Duroc gilt, Landrace pig, and Yorkshire sow. miRNAs were identified by Solexa sequencing followed by computer analysis. Hundreds of conserved and candidate novel miRNAs were identified. Our experiments have led to an increase in the number of porcine miRNAs identified. We also showed that almost all miRNAs exhibited isoforms of variable length and thus have potentially distinct function. Further more, a porcine miRNAs array was designed according to our Solexa sequencing results and the miRNAs in liver tissue of different pig breeds was analyzed using this array. Some differentially expressed miRNAs between different pig breeds were detected. These results should be particularly useful for studying the biological functions of miRNAs in pigs.

## Results

### Overview of the Solexa sequencing data

To increase the coverage of porcine miRNAs by Solexa sequencing, a small RNA library was constructed from the pooled porcine RNA samples collected from 16 tissues of young and adult pigs. Schematic illustration of the identification and character of porcine miRNAs was shown in Figure S1. The 3 different methods are described in detail in “material and methods” section. After Solexa sequencing, 4,821,809 raw reads were obtained from the small RNA library. The low-quality reads were removed, and the 3′ adaptor sequence was trimmed. The 5′ adaptor contaminants were also removed. Small RNA sequences ranging in size from 18–30 nt were retrieved from the raw data set. In total, we obtained 3,389,218 high-quality reads, which accounted for 70.30% of the total raw reads. To simplify the sequencing data, all identical sequence reads in the small RNA library were grouped and converted into sequence tags. Finally, we obtained 399,963 unique sequences with associated counts of individual sequence reads.

**Figure 1:**
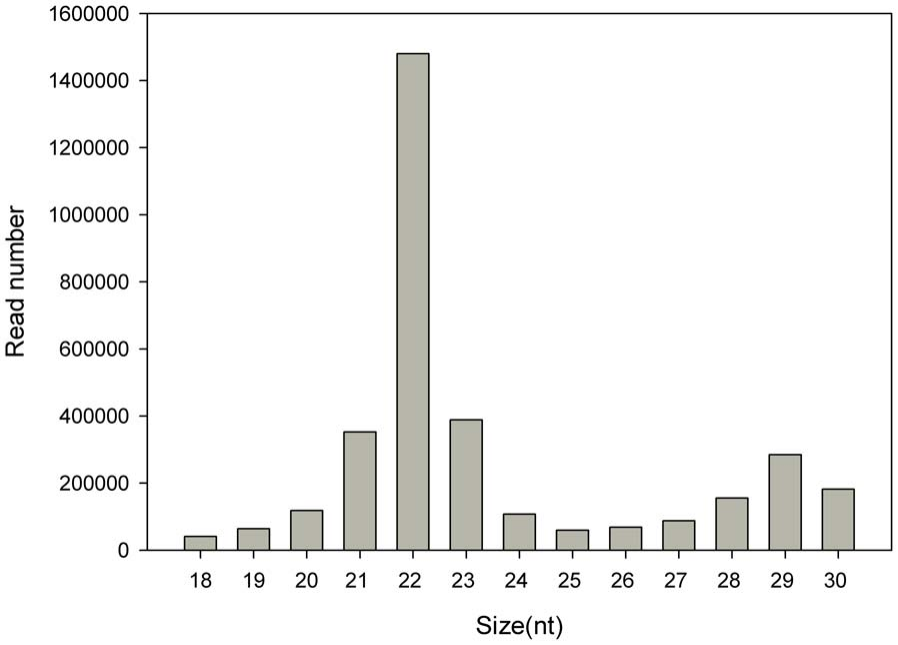
Sequence length distribution.

**Figure 2:**
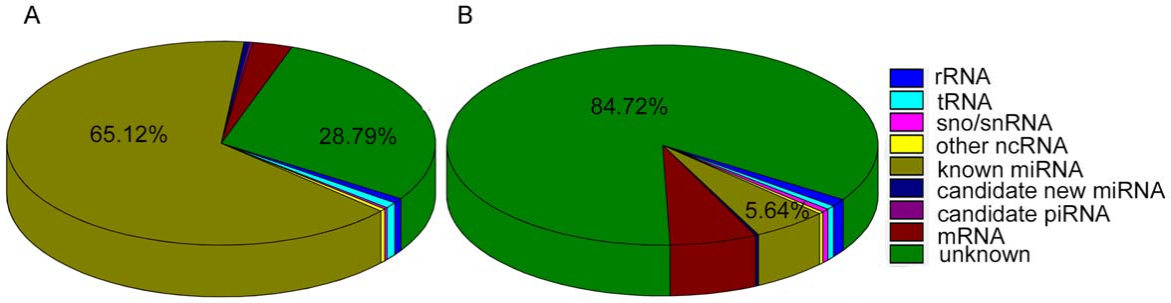
Count number distribution of different RNA class. A, Counted the Reads number; B. Counted the unique Tag number.

The size distribution of the reads is shown in Figure 1. The majority of the small RNAs were 21–23 nt in size. Sequences that were 22 nt in length accounted for 43.69% of total sequence reads, which is the typical size range for Dicer-derived products. The 21-nt size class was also dominant. The composition of the RNA classes is shown in Figure 2 (Detail see Table S14). The small class of cloned RNAs represents fragments of less abundant noncoding RNAs (rRNA, tRNA, small nuclear RNA, small nucleolar RNA, and piRNA), and this was identified by BLASTN searches against the Rfam and RNAdb databases [35]. A fraction also represents mRNA breakdown products from the pig. The remaining sequences formed the annotated porcine miRNAs, and these accounted for 65.12% of the total sequence reads in this small RNA library (Figure 2, A). These results indicated that the small RNA library was highly enriched in miRNA sequences. However, the sequence reads that were derived from annotated miRNAs represented only a relatively small fraction (5.64%) of the total number of unique sequence tags (Figure 2, B). The highest fraction of unique sequence tags (84.72%) was attributed to unknown RNAs (Figure 2, A). This indicated that the annotated miRNAs might be the most abundant class of small ncRNAs in different porcine tissues. There was a less abundant but much more diverse class of small RNAs that could represent other classes of small ncRNAs. This is consistent with the results of Glazov *et al.* (2008) who reported the identification of chicken embryo miRNAs using a Solexa deep sequencing approach [36].

### Identification of conserved and novel miRNA candidates by miRDeep

After removing the sequence reads with recognizable adapters, unique sequence reads of size ≥15 nt were analyzed using the miRDeep software to detect conserved and novel porcine miRNAs (method 1). This program uses a probabilistic model of miRNA biogenesis to score the compatibility of the position and frequency of sequenced RNA with the secondary structure of the miRNA precursor [37]. Through this method, 195 porcine miRNAs with high sequence identity (>95%) to known animal miRNAs (miRBase 16.0) were identified by miRDeep program (Table S1). The results also showed that 36 porcine miRNAs that had already been deposited in miRBase were also present in our deep sequencing data, including ssc-let-7c, ssc-miR-103, and ssc-miR-106a. Some miRNA-star sequences (miRNA*, the other arm of the stem) identified by miRDeep were present in high abundance in the pig. One example is ssc-miR-140*. This miRNA* sequence read number was 8483, which is much higher than that of ssc-miR-140 (the read number of miR-140 was only 4), indicating that miRNA* might be the functional form in pigs (Table S1).

Candidate novel miRNAs were simultaneously identified from deep sequencing data by miRNA precursor prediction using miRDeep. To determine whether these miRNA sequences are conserved in mammals, they were searched against the NCBI “non-redundant” database. Sequences that are parts of repetitive elements or annotated function RNAs were excluded. None of these miRNA sequences could find identity sequences in other species, indicating that these may be potential novel pig specific-miRNAs. Typically, miRNAs are 18–26 nt long, and 67 of these new miRNAs were identified and termed the “ssc-miR-new plus number” (Table S2). In addition, a class of miRNAs named “ssc-miR-large plus number,” which ranged in size from 27–30 nt, was also predicted by miRDeep (Table S2). The Vienna package and Randfold application were applied to the miRDeep package to confirm whether these new miRNAs could form typical secondary structures. Another tool, the mfold software, was also used [38]. The mfold results showed that some of the new miRNAs identified by miRDeep could not form typical secondary structures, and these were therefore discarded. The secondary structures of the remaining miRNAs are shown in Table S3. The folding free energy (ΔG) of each structure was given by mfold; the results indicated that their ΔG value was lower than -17 kcal/mol. The genomic locations of the novel candidate miRNAs on the WGS sequence from the NCBI database were generated by miRDeep and are listed in Table S4. Subsequently, the chromosome location on the pig draft genomic sequence from the Sanger database was determined using the BLASTN software (Table S9). As an example, ssc-mir-new2, ssc-mir-new5, and ssc-mir-new35 were found on chromosome X.

### Identification of conserved miRNA candidates by the BLASTN method

To determine whether known miRNAs from other species are also present in our porcine deep sequencing data, BLAST matching of known animal mature miRNA sequences (miRBase16.0) to our sequence tags was performed using the command-line blastall program [13], with the expect value set at 1 (to be stringent when matching the short lengths of known miRNA sequences) and using the BLASTN nucleotide program (method 2). The results showed that 473 unique sequence tags had perfect matches to known animal miRNAs (Table S5), and 343 had sequence variations to known animal miRNAs (Table S6). When comparing miRNAs across different species, we found minor differences in the nucleotide composition of the miRNAs of some species. Therefore, the number of conserved candidate porcine miRNAs is listed in the table more than once. For example, mature miRNA let-7b from *Pan troglodytes* and *Xenopus tropicalis* differed by 2 nucleotides. We found 2 unique porcine tag sequences that exactly matched these. The read number of the sequence that matched ptr-let-7b was 88,532. However, there were 4 other sequences that matched xtr-let-7b (Table S5). Consequently, it was difficult to determine whether one sequence belonged to the true let-7b miRNA in pig or whether they were different isomiRs, or could be due to sequencing error. To simplify the dataset, Sequences which under the same miRNA name was classified as one miRNA species. As a result, 416 porcine miRNAs were found by using BLASTn method.

### Implementation of the miRDeep program to search for conserved mature miRNAs from porcine genomic sequences

To determine whether other reported animal miRNAs could be sequenced and captured by Solexa sequencing in our study, miRDeep software was re-adopted to predicate porcine miRNAs (method 3). This approach is similar to method 1, but using known animal mature miRNAs (miRBase 16.0) instead of porcine Solexa sequences. To test the ability of miRDeep detect known miRNAs, all known porcine mature miRNA sequences that had already been deposited in the miRBase database were used. As a result, 201 porcine homolog (identity>95%) miRNAs were detected (Table S7). The corresponding porcine miRNA precursor sequences, secondary structure and genome location were listed in Table S8. Some known porcine miRNAs were also successfully detected by miRDeep, such as ssc-let-7c, ssc-miR-103, and ssc-miR-122. The results indicated that miRDeep can be used not only to analyze high-throughput sequence data but also to predict miRNAs from genomic sequences.

### Sequence variations in miRNAs

**Figure 3:**
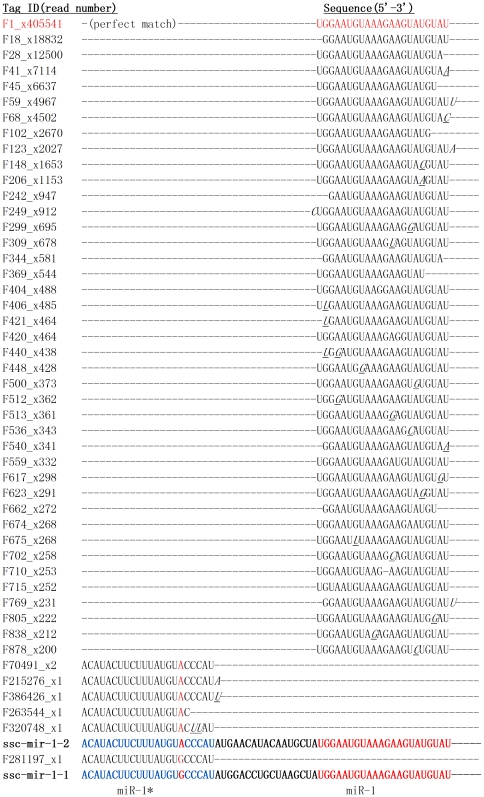
Example of high frequency of miRNA sequence variations (isomiRs). Shown are the unique sequences and number of times this sequence was detected matching the pre-miRNA sequence of ssc-miR-1. The most frequent occurring miR-1 sequence is in accordance with the miRBase reference sequence. An example of a miR-1 isomiR not matching the genome is shown in the italic part of the figure.

In our Solexa library, porcine miRNAs frequently exhibited variation from their “reference” sequences, producing multiple mature variants that we hereafter refer to as isomiRs. The miRNA* sequence and its isomiRs were also observed in this library. Evidence of isomiRs of a similar nature were detected in a limited sequence analysis using a linker based miRNA cloning approach in our previous studies from a single RNA pool [30], suggesting that isomiRs are not due to artifacts created from massively parallel sequencing (Figure. 3). All isomiRs are listed in Table S10. In total, we found 23822 isomiRs for a total of 459 sequenced miRNAs and miRNA*s (Table S10). The kinds of different isomiRs for a given miRNA range from one to 1051(ssc-miR-22a and ssc-let-7b) and 56 miRNAs exhibit more than one hundred kinds of isomiRs. There are 64 miRNAs that have no isomiRs. In 77 cases, this most abundant sequence did not correspond exactly to the current porcine miRBase 16.0 reference sequence (Table S10). For example, we found that the size of ssc-miR-29b in our library is 23 (read number is 88), while the size of miR-29b in miRBase is 20 (read number is 11). The size of another miRNA ssc-miR-505 in our library is 18 (read number is 6), while the size of miR-505 in miRBase is 20 (read number is only 1). It seems that under the different physiological conditions, the process length of miRNAs may vary, which produce different isomiRs. This also suggests either that the relative abundance of isomiRs may vary across different studies or that the original submission of this miRNA to miRBase was incorrect. Following the most recent update to miRBase, far more of the most abundant sequences in our library corresponded with the updated miRBase reference, pointing toward the latter explanation.

### Determination of tissue expression patterns of precursor and mature miRNAs by Northern blotting

**Figure 4:**
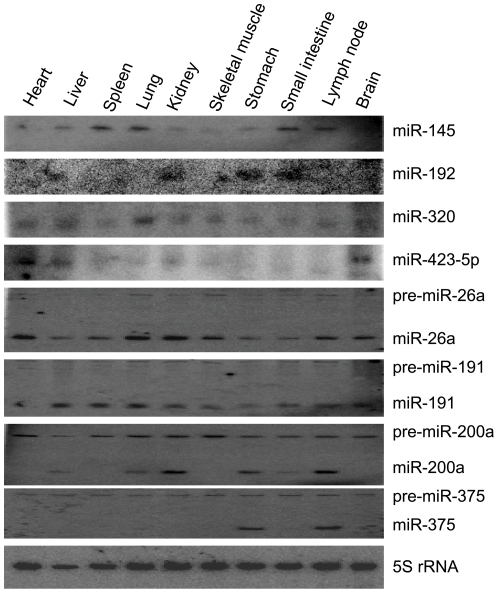
Expression profiles of conserved porcine miRNAs by Northern Blotting. Total RNA isolation from porcine heart, liver, spleen, lung, kidney, skeletal muscle, stomach, small intestine, lymph node and brain, 5S rRNA served as a loading control. Hybridization signals were detected for pre-miRNAs or/and mature miRNAs.

miRNA expression patterns are generally examined to obtain a better understanding of the physiological functions of these molecules. Moreover, *in vivo* expression analysis provides additional evidence for the existence of *bona fide* miRNAs. To experimentally confirm and validate the results obtained from the Solexa library, we examined the expression patterns of conserved miRNAs. These miRNAs were randomly selected and experimentally verified by Northern blotting hybridization in 10 normal pig tissues, i.e., the heart, liver, spleen, lung, kidney, skeletal muscle, stomach, small intestine, lymph node, and brain. Antisense RNA probes that were designed to hybridize to mature miRNA and 5S rRNA are listed in Table S17. Using these probes, miR-145, miR-192, miR-320, and miR-423-5p showed signals that were approximately 22 nt in size, while the other probes detected not only the mature form but also the approximately 75-nt precursors on the same blots (Figure 4). The results also showed that the tissue expression patterns or the expression levels of the precursor and mature forms differed. For example, the precursor forms of mir-375 and mir-200a were ubiquitously expressed in 10 tissues, while mature miR-375 was only detected in the stomach and lymph node. Moreover, miR-200a was undetectable in the heart, spleen, skeletal muscle, and brain. Although the expression patterns of the precursor and mature forms of miR-26a and miR-191 were consistent across tissues, the expression levels of the precursor forms of mir-26a and mir-191 were much lower than those of their mature forms. The expression patterns of some miRNAs indicated selective expression in some tissues, for example, miR-192 was only expressed in the kidney, stomach, and small intestine at low levels. miR-200a was highly expressed in the kidney, lymph node, and stomach and was moderately expressed in the other tissues. miR-320 was abundantly expressed in the lungs but was expressed in trace amounts in other tissues. miR-145 was expressed at high levels in the spleen, lung, and small intestine, and miR-26a was relatively strongly expressed in the heart, lung, and kidney (Figure 4).

### Stem-loop RT-qPCR

**Figure 5:**
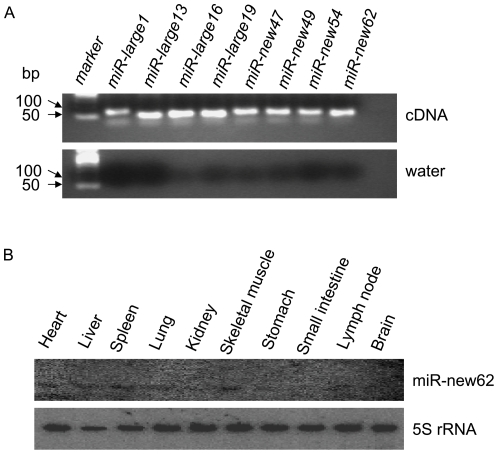
Validation of candidate novel porcine miRNAs. A. Validation of novel miRNAs by Stem-loop RT-PCR; B. The expression profile of ssc-miR-62 was detected by Northern blotting. Total RNA isolation from porcine heart, liver, spleen, lung, kidney, skeletal muscle, stomach, small intestine, lymph node and brain, 5S rRNA served as a loading control. Hybridization signals were detected for mature new miRNA.

The stem-loop RT-qPCR assay was used to specifically detect mature miRNAs, as reported previously [39]. To validate novel candidate miRNAs found by Solexa sequence and miRDeep approach, thirty-five miRNAs were randomly selected to perform end-point RT-PCR (Table S17). The results showed that PCR products of the predicted size could be obtained using template cDNA, and the control without the template (water) gave no signals (Figure 5). Using this approach, 8 candidate novel miRNAs (ssc-miR-large1, ssc-miR-large13, ssc-miR-large16, ssc-miR-large19, ssc-miR-new47, ssc-miR-new49, ssc-miR-new54, and ssc-miR-new62) could be amplified and their specific bands could be detected by stem-loop RT-PCR (Figure 5, A). No specific band was detected for the other selected miRNAs. Among these, only one miRNA, i.e., ssc-miR-new62, was detected and expressed in the skeletal muscle and spleen and in trace amounts in other tissues, as determined by Northern blotting. The results confirmed that this novel miRNA exists in pigs (Figure 5, B). The signals of several candidate novel miRNAs were not detected by the above Northern blotting method, and it is possible that these may have relatively low expression levels in the selected tissues.

### Detection of differential expression in liver between fatty and lean pigs using a self-designed mammalian miRNA microarray

**Figure 6:**
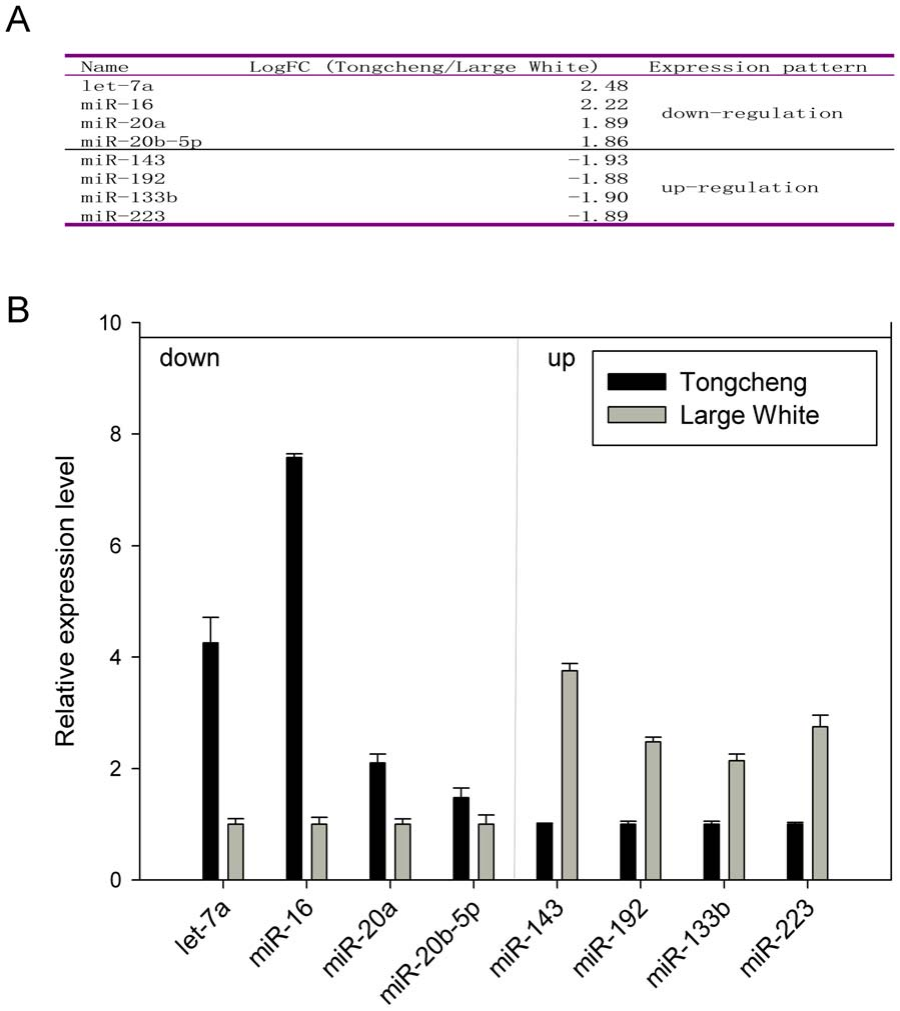
Validation of the microarray results using Real-time PCR method. Expression levels of eight miRNAs were detected by microarray (A) and Real time PCR (B).

All porcine miRNAs which obtained using the above methods were listed in Table S12. Subsequently, we designed a miRNAs array using all porcine miRNAs that are listed in this Table and miRNAs of other species in miRBase. Using this porcine miRNA array, 123 miRNAs were detected expression in porcine liver tissues (with a positive rate 22.5%), which including 19 new miRNAs detected by Solexa deep sequencing. The top 10 miRNAs which highly expressed in the liver were miR-720, miR-122, miR-923, miR-1826, miR-1260, miR-large1, miR-new51, miR-new34, mirR-894 and mirR-new36. Four among them were new miRNAs which detected by Solexa deep sequencing. According to our microarray result, we found there were 58 miRNAs differentially expressed (LogFC≥|±1.5|, P<0.05) between the Tongcheng and Large White pig breeds (Table S13). In order to verification the differential expressed miRNAs, four down-regulated (let-7a, miR-16, miR-20a, miR-20b-5p) and 4 up-regulated miRNAs (miR-143, miR-192, miR-133b, miR-223) were randomly selected and analyzed using Real-time PCR method (Figure 6, A). Then, the Pearson correlation coefficient of the Real-time PCR assay and the microarray assay was conducted and the value in down-regulated miRNA dateset is 0.7955, in up-regulated miRNAs dateset is 0.8889 (Figure 6, B). This result indicated that there was good correlation between the microarray analysis and the qPCR analysis. These results indicated that our self-designed mammalian miRNA microarray was suitable for porcine miRNAs analysis. Moreover, these differentially expressed miRNAs may play important roles in the liver between fat and lean type pig breeds.

## Discussion

**Figure 7:**
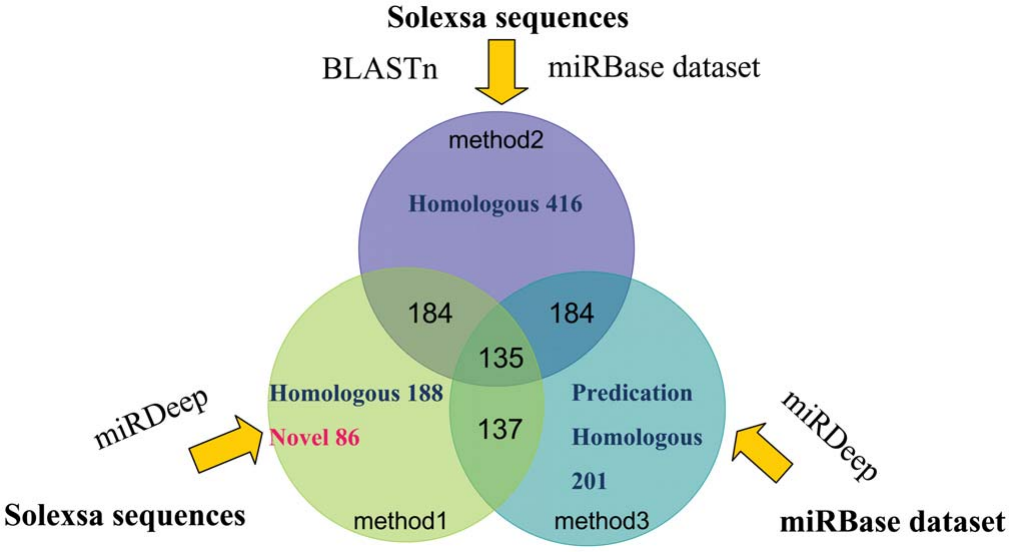
Comparison of miRNA sequences identification with different Methods.

The domestic pig (*Sus scrofa*) is an important economic animal for meat production and as a suitable model organism for comparative genomics and biomedical studies [22], [23]. To identify more pig miRNAs, especially pig-specific novel miRNAs, a small RNA cDNA library from a mixture of different porcine tissues was constructed for deep sequencing using the Solexa sequencing technique. Subsequently, we used a combination of the miRDeep algorithm and the homology search method to obtain 437 conserved miRNAs and 86 candidate novel pig-specific miRNAs. All porcine miRNAs which obtained by using different methods were listed in Table S12. The genomic locations of the conserved miRNAs on the WGS sequence from the NCBI database were generated by method 1 and method 3 was listed in Table S8. Then, the chromosome location on the pig draft genomic sequence from the Sanger database was determined using the BLASTN software (Table S9). Among these, 188 conserved and 86 candidate novel miRNAs were found from the Solexa sequencing data by method 1, and 416 miRNAs were identified from the Solexa deep sequencing data using the method 2. Moreover, 137 miRNAs were predicted in the pig genome using the method 3 (Figure 7). As shown in Figure 7, 184, 184, and 137 miRNAs overlapped across the three different methods, respectively, and 135 miRNAs were simultaneously identified by the three methods. Comparison of the results of porcine miRNA sequence identification by different approaches revealed that some of the miRNA sequences have minor differences, which could be detected by using different methods. Due to the close phylogenetic distance between the pig and human, the majority of the sequence reads corresponded to previously annotated humans miRNAs, and only a few potential novel pig-specific miRNAs were obtained.

In this study, we used direct sequencing and computer prediction methods to identify porcine miRNAs. However, we found that each method has certain limitations. The miRDeep software is designed for miRNA annotation from the new high-throughput sequencing data generated by Solexa, 454, etc. This program first maps the sequences to the genome and then indicates whether a sequence is an miRNA based on the structural features of Dicer enzyme processing and the frequencies of sequence reads [37]. In method 1, the main advantage of using miRDeep software is that it can simultaneously identify conserved and new miRNAs, and the accuracy is good because this software uses a probabilistic model of miRNA biogenesis to score the compatibility of the position and the frequency of the sequenced RNA with the secondary structure of the miRNA precursor. One disadvantage is that some new miRNAs identified by miRDeep are actually false positives. Thus, new miRNAs should be double checked with “mfold” software to confirm their identity. Another problem is that the complete genomic sequence of pigs is not available, and many miRNAs that do not align to the pig genome cannot be identified by miRDeep. Therefore, in addition to miRDeep, we used BLASTn program (method 2) to identify pig miRNAs. In general, miRNAs are conserved among species, and homology search can help in detecting miRNAs by comparing the Solexa sequences to miRNAs in miRBase even when the complete genomic sequence of the species is not known. However, the drawback of this method is that some miRNAs match the same miRNA in different species, and there are one or two base pair differences among the miRNAs. Therefore, it may be difficult to determine whether the sequences that match these miRNAs are sequence error or whether they are isomR. For example, porcine F37976_x4, F1_x405541, and F500_x373 from the Solexa sequences matched dya-miR-1, ptr-miR-1, and rno-miR-1, respectively. These three miRNAs have sequence differences, so F37976_x4, F1_x405541, and F500_x373 may be isomiR of miR-1 in the pig. However, miRNA end nucleotides are difficult to identify, irrespective of whether miRDeep or homolog search is used. This is especially true for the 5′ end sequences, which are important because they contain the “seed” region of the miRNAs. Taken together, miRDeep can provide accurate annotations of miRNAs from short sequences. When the detected miRNA from miRDeep has a homolog in the model species, the end nucleotides can be determined according to the homologous miRNAs. To find other porcine miRNAs which may not have been captured by using Solexa sequencing, miRDeep program was re-adopted to predicate miRNA in porcine genome (method3), the main advantage of using this method is that some tissues specific and/or developmental specific miRNAs, which not detected by Solexa sequencing may be found *in silico* method.

Analyses of the 437 conserved and 86 candidate novel miRNAs identified by different methods showed that 116 miRNAs are 21 nt long (22.2%), 212 miRNAs are 22 nt long (40.5%), and 99 are 23 nt long (18.9%). This is in agreement with the library sequence distribution analysis results, which showed that sequences that were 21–23-nt in length had the highest frequency. A total of 66 piwiRNAs were obtained by homology search (Table S11). Some rRNA, tRNA, and sno/snRNAs were also found in the library. The proportion of miRNAs was 5.64% in all of the nonredundant sequences in the library (Table S14). The percentage of miRNAs was 65.12% when the redundant sequences were counted. Most of the sequences (91.01%) had more than 1 read (Table S15). Some sequences could not be annotated, which may be due to the incomplete nature of the pig genomic sequence.

To show the sequence variations between our library and 212 porcine miRNAs in miRBase (Release: 16.0) [24], [25], [26], [27], we found that 115 miRNAs identified from this study had perfect match with each other, and 77 had 1 or 4 base variations at the length of terminus. Among the sequence identical group, we found a candidate novel miRNA miR-new66 had perfect match with the known porcine miRNA ssc-miR-676-3p (miRBase 16.0). Moreover, two of the 86 candidate novel pig-specific miRNAs were also identified in a porcine skeletal muscle library in our lab [30]. A total of 88 of the 296 miRNAs identified by Huang *et al.* (2008) had perfect matches in our library [28]. Using the Solexa sequencing and microarray techniques, the expression of 332 intestinal miRNAs was uncovered [34]. Comparison of these revealed that a total of 139 of the 332 miRNAs identified by Sharbati *et al.* (2010) had perfect matches in our library, including 8 novel pig-specific miRNAs, such as miR-new3, miR-new6, miR-new10, miR-new16, miR-new19, miR-new28, miR-new56, miR-new66. In addition, some of them were validated by the stem-loop RT-PCR method (Figure 6). These findings further support the notion that they may exist in the pig. While our manuscript was prepared, one paper was published on PLoS ONE in which hundreds of miRNAs were identified in the pig by Solexa sequence[40]. When we compared this data with ours, we found that although those researchers performed a relatively comprehensive search for porcine miRNAs on ten small RNA sequencing libraries prepared from a mixture of tissues obtained in different ages, only 272 had perfect matches with the miRNAs identified in our study. There were 83 miRNAs had 1 or 2 base variations. Compared with all the reported porcine miRNAs, we found that 230 miRNAs, including candidated novel miRNAs, which identified from this study had perfect matches with each other, and 120 had 1 or 2 base variations at the length of terminus. In other words, 112 conserved and 61 candidate novel porcine miRNAs were first reported in our study. We also compared the identities of porcine miRNAs from this study with known human, mouse, and rat miRNAs (miRBase 16.0). A total of 414 porcine miRNAs in our study matched (identity>95%) with their human, mouse, and rat counterparts. This result showed that porcine miRNAs had high homology to miRNAs from human and rodent, which was in agreement with previous reports [24].

Several studies showed that isomiRs may function in the animal [41], [42]. Our analysis revealed a large number of isomiRs, derived from almost all detected miRNAs, in total, we detected 23822 isomiRs in our library, which greatly exceeded and extended previous reports. Further analysis revealed two major classes of variants or isomiRs(Table S10). Most isomiRs showed variability at their 5′ and/or 3′ ends, likely resulting from variations in the pre-miRNA secondary structures that result in variable cleavage sites for Dicer1 or Drosha. An example is given in Figure 3, demonstrating sequenced isomiRs for miR-1, which indicated that the majority of porcine miRNA nucleotide variants resulted from post-transcriptional modifications. The presence of isomiRs may have various functions and require further research.

The seed sequence is formed by seven or eight nucleotides of the mature miRNA, such seed regions are mostly conserved among miRNAs of the same family [5]. Here we used 8mer sites (an exact match to positions 2–8 of the mature miRNA) as parameter to classify different miRNA families, especially the candidate novel miRNAs. As a result, all the mature miRNAs can be classified into 300 different miRNA families (Table S16). We further compared the largest conserved miRNA families among different species, the let-7/98 family. This family is the first miRNA family to be reported. There is a very low rate of nucleotide change among its members. The human let-7/98 family contains 9 members, i.e., hsa-let-7a/b/c/d/e/f/g/i and hsa-miR-98. The rat let-7/98 family has 8 members (let-7g is not included). We found that the porcine let-7/98 family has 12 members including the 9 human homologous and ssc-let-7h/j/k miRNAs (Table S16). By analysis of candidate novel miRNAs, we found some of them can be classified into the conserved miRNA families, for example, the seed sequence of miR-new42 is exactly the same as the miR-28 and miR-708's.The 5′ end sequences are crucial for classifying the miRNA families, and a change in even one base pair can result in failed classification of members of the same family. Based on these results, we concluded that our Solexa library is of high quality and allows easy identification of pig miRNAs.

Using our porcine miRNA-array, 123 miRNAs were detected expression in porcine liver tissues, including 19 new miRNAs found by Solexa sequencing. Among the top 10 expressed miRNAs, miR-122 has been reported as an abundant liver-specific miRNA [43], [44], other highly expressed miRNAs were firstly reported in this study. There were 58 miRNAs differentially expressed between the Tongcheng and Large White breeds. Let-7a was the most highly expressed let-7 family member, it has been confirmed as a negative regulation signal transducer and activator of transcription 3 (STAT3), which is associated to hepatocellular proliferation and hepatocarcinogenesis [45]. TGFbeta-R1 also had been confirmed as a target gene of let-7c, which participated in liver development. These results indicated that let-7 family members play important roles in liver development [46]. Let-7 miRNAs were differentially expressed between Tongcheng and Large White pigs, indicating the gene expression regulation at posttranscriptional level in liver is different between lean and fatty pigs at the same body weight. miR-16 was shown to regulate liver cell line HepG2 apoptosis by targeting BCL2 gene [47], is also detected differential expression between the two breeds. Other differentially expressed miRNAs have no reports at present. Our result indicated that these differentially expressed miRNAs between different breeds may play important roles related to liver development, liver cell apoptosis and energy metabolism. These results also indicate our miRNA array is an appropriate tool for porcine miRNAs expression detection.

## Materials and Methods

### Tissue sample collection and RNA isolation

The following tissues were collected and used to generate small RNA libraries: the heart, liver, longissimus dorsi muscle, and stomach from a one-week-old Duroc gilt; the spleen, lung, kidney, small intestine, inner fat, thymus, mesenteric lymph node, submandibular lymph node, and testicle from a four-month-old Meishan boar. The endometrium and placenta from a pregnant Yorkshire sow, and a 33-day-old whole embryo from a Landrace sow. Samples were immersed in liquid nitrogen immediately after collection and then stored at −80°C. Total RNA was isolated from porcine tissues using the Trizol reagent (Invitrogen, Carlsbad, CA, USA), according to the manufacturer's instructions; the samples were then pooled. The quality of total RNA was checked on an Agilent 2100 Bioanalyzer system. RNA samples were stored at -80°C until further use. All research involving animals were conducted according to the regulation (No. 5 proclaim of the Standing Committee of Hubei People's Congress) approved by the Standing Committee of Hubei People's Congress, and the ethics committee of Huazhong Agricultural University, P. R. China. The approved permit number for this study is “HBAC20091138”.

### Small RNA library construction and sequencing

For small RNA library construction and deep sequencing, approximately 20 µg of pooled RNA was used. The sequencing was performed as follows: RNA was purified by polyacrylamide gel electrophoresis (PAGE) to enrich molecules in the range of 16–30 nt, and proprietary adapters were ligated to the 5′ and 3′ termini of the RNA. The samples were used as templates for cDNA synthesis. The cDNA was amplified using the appropriate PCR cycles to produce sequencing libraries that were subjected to Illumina Genome Analyzer's proprietary sequencing-by-synthesis method. Sequencing was carried out at the Beijing Genomics Institute (BGI, China).

### Sources of sequences and software

Before performing the sequence annotation, some necessary sequence data and software were retrieved from known websites. Preliminary sequence assemblies for pig chromosomes can be viewed in Pre Ensembl, and the assembled sequence was downloaded from ftp://ftp.sanger.ac.uk/pub/S_scrofa/assemblies/PreEnsembl_Sscrofa7/(updated, 14th July 2008). The raw sequence data of pigs, which is in the form of traces at the NCBI Ensembl Trace Archive, was downloaded from ftp://ftp.ncbi.nih.gov/pub/TraceDB/sus_scrofa/. The ncRNA data was downloaded from the Rfam 9.0 database (http://rfam.janelia.org/). Pig mRNAs were retrieved from the NCBI Nucleotide database (http://www.ncbi.nlm.nih.gov/) using the input “(pig mRNA)” AND “Sus scrofa [porgn:_txid9823].” Known miRNAs/miRNA hairpins were obtained from miRBase (Release15.0) (http://microrna.sanger.ac.uk/sequences/). The miRDeep package was downloaded from http://www.mdc-berlin.de/rajewsky/miRDeep. In addition to Perl (available at http://www.perl.com/), the Vienna package (available at http://www.tbi.univie.ac.at/RNA), Randfold application (http://bioinformatics.psb.ugent.be/software/details/Randfold), and NCBI BLAST package (http://www.ncbi.nlm.nih.gov/BLAST/download.shtml) that required dependencies were downloaded from their respective sites. The piRNA data was retrieved from RNAdb (http://jsm-research.imb.uq.edu.au/rnadb/default.aspx).

### Data analysis

Individual sequence reads with the base quality scores were produced by Solexa. Low-quality reads were strictly removed from the raw reads. After trimming the 3′ adaptor sequence and removing the 5′ adaptor contaminants, all identical sequences of sizes ranging from 18–30 nt were counted and eliminated from the initial data set. The resulting unique sequences with associated read counts are referred to as sequence tags. All unique sequences were used to search the ncRNA data (Rfam) with BLASTN to remove non-miRNA sequences (rRNA, tRNA, etc.).Schematic illustration of the identification and character of porcine miRNAs was shown in Figure S1. Details of the three methods are as follows: Method 1, miRDeep program was used to identify porcine miRNAs from large pool of unique sequenced transcripts from our Solexa deep sequencing. All candidate Solexa RNA sequences between 18 nt and 30 nt were aligned against raw sequence data (NCBI Trace Archive) using the miRDeep software with the default parameter. Subsequently, the miRDeep miRNA precursors obtained from the short read library were searched against the pig genome (Ensembl Trace Archive) to determine the chromosome location. Furthermore, putative novel miRNAs were searched against the NCBI NR database to determine whether these miRNAs exist in other species. These miRNA precursor sequences were then used for fold-back secondary structure prediction with the mfold program. Method 2, all unique candidate Solexa RNA sequences were re-queried against already known animal mature miRNAs (from the latest miRBase database, release 16.0) by using BLASTn software to the further identification of conserved porcine miRNAs. When the BLAST program was run, the parameter Expect value was set at 0.001 to improve the reliability. To identify more miRNAs in the pig genome, method 3 was re-adopted miRDeep program to predicate the porcine miRNAs, which using all known animal mature miRNAs (miRBase) instead of our Solexa reads aligned against raw pig genome sequence data. Finally, porcine miRNAs identified by the three above-described methods were combined. The putative origins of the remaining sequences were identified by BLASTN search against the pig mRNA data from NCBI.

Sequences in the 26–30 nt size range, which were not annotated as miRNA, mRNA, rRNA, tRNA, etc., were used to search the piRNA data (RNAdb) with BLASTN and to check whether the small RNA library that we had generated contains piRNAs. Only perfect matches were classified as candidate piRNAs.

### Northern blotting

Small RNA blot analysis was performed to determine the expression patterns of conserved and new miRNAs. Total RNA (20 µg) was resolved on a 15% denaturing polyacrylamide gel. RNA was electrophoretically transferred to Hybond™-N+ (Amersham) membranes, and the membranes were UV crosslinked and baked for 30 min at 80°C. DNA oligonucleotides complementary to small RNA sequences were end-labeled with γ-32P-ATP using T4 polynucleotide kinase (NEB), and these oligonucleotides were used as the probes (Table S17). The blots were pre-hybridized for at least 30 min and hybridized overnight with the PerfectHyb™ buffer (Toyobo, Osaka, Japan) at 42°C. The blots were then washed with 2× SSC/0.1% SDS buffer three times at 42°C and then autoradiographed. The blots were visualized in a FLA 9000 biomolecular imager and analyzed with the Multi Gauge software (Fujifilm, Tokyo, Japan). Pig 5S rRNA served as the loading control.

### Stem-loop RT-PCR

Real-time PCR was performed using a standard SYBR Green PCR kit (Toyobo, Japan) in the BioRad iQ5 Real-Time PCR Detection System according to the manufacturer's instructions. Stem-loop RT primers were designed according to Chen et al (2005) and listed in the Table S17.The reverse transcriptase reaction using Stem-loop primers was performed according to the protocol of our previous studies[30].

### New mammalian miRNA-array design

In order to develop new tools to use the porcine miRNAs isolated through Solexa deep sequencing in biological studies, a new mammalian miRNA-array was designed according to the miRNAs submitted in miRBase and our porcine miRNAs. This miRNA-array contains 1551 specific miRNA probes, including 988 miRNAs of human, 350 miRNAs of rat, 627 miRNAs of mouse and 546 miRNAs of pig, respectively. U6 and tRNA probes were designed for internal positive control. Moreover, eight artificial probes of 30 nt named as Zip5, Zip13, Zip15, Zip21, Zip23, Zip25, Y2 and Y3 were used as external control. 50% DMSO were used as negative control. The probes were concatenated up to a length 40 nt (3′ -end miRNA plus 5′ -end PolyT) and attached to the slide surface Via a C6 5′-amino-modifier. The probes were spotted to the 75 mm×25 mm chip at a concentration of 40 µm using SmartArray™ (CapitalBio Corp., Beijing, China) and the HEX labeled probes were used as positive control for quality of the spotting. Each probe was printed in triplicate on the miRNA-array.

### Porcine miRNAs expression detection

MiRNAs expressed in liver were detected using our miRNA-array. Total liver RNAs from three Tongcheng pigs and three Large White pigs at about 25 kg body weight were extracted using TRIzol reagent (Invitrogen, Carlsbad, CA, USA). Subsequently, the low-molecular-weight RNA isolated using PEG reagent and labeled with Cy3 (Dharmacon, USA) according to the previous studies [48], [49]. For slide hybridization, 5 µg Cy3 labeled RNA was dissolve in 20 µl hybridization mixture (15% formamide; 0.2%SDS; 3×SSC; 50×Denhardt's) and hybridized overnight. Then, slides were washed in solution of 0.2% SDS, 2×SSC at 42°C for 4 minutes, and in 0.2×SSC for 4 minutes at room temperature. The slides were scanned with LuxScan 10K/A scanner (CapitalBio Company, China) and the raw pixel intensities were analyzed by LuxScan3.0 software (CapitalBio Company, China). The median pixel intensities that failed to exceed the negative control value by more than two standard deviations (Signal<negative mean+2 *SD*) were considered as negative hybridization. One miRNA was considered to be detected when it has positive signal at least at two miRNA-arrays.

### Statistical analysis of microarray data

The normexp and quantile methods in limma package [50] were used to perform the background correction and normalization between arrays, respectively. After removing all control spots, 1594 miRNAs were fitted with linear model. Expression differences were compared between the two groups (Tongcheng and Large White pig) using standard t-test, which *P*-values were adjusted by using Benjamini and Hochberg's method.

## Supporting Information

Figure S1 Illustration of high-throughput identification of porcine microRNAs by deep sequencing.
(TIF)

Table S1 Porcine microRNAs were identified by miRDeep program.
(XLS)

Table S2 Putative novel porcine microRNAs which size range from 18–30 nt were identified by miRDeep program.
(XLS)

Table S3 Stem-loop structures of putative novel microRNA precursors.
(XLS)

Table S4 Putative novel microRNAs location on the WGS sequence from NCBI database.
(XLS)

Table S5 Unique sequences with a perfect-match to known animal microRNA (using BLAST method search against miRBase 16.0).
(XLS)

Table S6 Unique sequences with not perfect-match to known animal microRNA (using BLAST method search against miRBase 16.0).
(XLS)

Table S7 Porcine microRNAs were predicated by miRDeep program.
(XLS)

Table S8 Conserved porcine microRNA on the WGS sequence from NCBI database.
(XLS)

Table S9 Chromosome location of putative novel and conserved microRNAs (Sanger database).
(XLS)

Table S10 isomiR_breakdown.
(XLS)

Table S11 Unique sequences with a perfect-match to known piwiRNA (using BLASTn method search against RNAdb2.0).
(XLS)

Table S12 MicroRNA sequences identified using the three different methods.
(XLS)

Table S13 Differentially expressed miRNAs in the liver tissue of Tongcheng pig and Large White.
(XLS)

Table S14 Species of RNA and count number.
(XLS)

Table S15 Singleton vs. multiple sequence tags.
(XLS)

Table S16 microRNA families.
(XLS)

Table S17 Probes for hybridization to microRNAs and stem-loop primers for RT-PCR.
(XLS)

## References

[1] Bartel DP (2004). MicroRNAs: genomics, biogenesis, mechanism, and function.. Cell.

[2] Lee RC, Feinbaum RL, Ambros V (1993). The C. elegans heterochronic gene lin-4 encodes small RNAs with antisense complementarity to lin-14.. Cell.

[3] Reinhart BJ, Slack FJ, Basson M, Pasquinelli AE, Bettinger JC (2000). The 21-nucleotide let-7 RNA regulates developmental timing in Caenorhabditis elegans.. Nature.

[4] Pasquinelli AE, Reinhart BJ, Slack F, Martindale MQ, Kuroda MI (2000). Conservation of the sequence and temporal expression of let-7 heterochronic regulatory RNA.. Nature.

[5] Lewis BP, Burge CB, Bartel DP (2005). Conserved seed pairing, often flanked by adenosines, indicates that thousands of human genes are microRNA targets.. Cell.

[6] Carrington JC, Ambros V (2003). Role of microRNAs in plant and animal development.. Science.

[7] Wu L, Fan J, Belasco JG (2006). MicroRNAs direct rapid deadenylation of mRNA.. Proc Natl Acad Sci U S A.

[8] Yekta S, Shih IH, Bartel DP (2004). MicroRNA-directed cleavage of HOXB8 mRNA.. Science.

[9] Lee Y, Ahn C, Han J, Choi H, Kim J (2003). The nuclear RNase III Drosha initiates microRNA processing.. Nature.

[10] Lee Y, Kim M, Han J, Yeom KH, Lee S (2004). MicroRNA genes are transcribed by RNA polymerase II.. Embo J.

[11] Lagos-Quintana M, Rauhut R, Yalcin A, Meyer J, Lendeckel W (2002). Identification of tissue-specific microRNAs from mouse.. Curr Biol.

[12] Brennecke J, Hipfner DR, Stark A, Russell RB, Cohen SM (2003). bantam encodes a developmentally regulated microRNA that controls cell proliferation and regulates the proapoptotic gene hid in Drosophila.. Cell.

[13] Ambros V, Bartel B, Bartel DP, Burge CB, Carrington JC (2003). A uniform system for microRNA annotation.. Rna.

[14] Xiao C, Rajewsky K (2009). MicroRNA control in the immune system: basic principles.. Cell.

[15] Baek D, Villen J, Shin C, Camargo FD, Gygi SP (2008). The impact of microRNAs on protein output.. Nature.

[16] Bandiera S, Hatem E, Lyonnet S, Henrion-Caude A microRNAs in diseases: from candidate to modifier genes.. Clin Genet.

[17] McManus MT (2003). MicroRNAs and cancer.. Semin Cancer Biol.

[18] Lai EC (2002). Micro RNAs are complementary to 3′ UTR sequence motifs that mediate negative post-transcriptional regulation.. Nat Genet.

[19] Forman JJ, Coller HA The code within the code: MicroRNAs target coding regions.. Cell Cycle.

[20] Lytle JR, Yario TA, Steitz JA (2007). Target mRNAs are repressed as efficiently by microRNA-binding sites in the 5′ UTR as in the 3′ UTR.. Proc Natl Acad Sci U S A.

[21] Vasudevan S, Tong Y, Steitz JA (2007). Switching from repression to activation: microRNAs can up-regulate translation.. Science.

[22] Lunney JK (2007). Advances in swine biomedical model genomics.. Int J Biol Sci.

[23] Critser JK, Laughlin MH, Prather RS, Riley LK (2009). Proceedings of the Conference on Swine in Biomedical Research.. Ilar J.

[24] Wernersson R, Schierup MH, Jorgensen FG, Gorodkin J, Panitz F (2005). Pigs in sequence space: a 0.66X coverage pig genome survey based on shotgun sequencing.. BMC Genomics.

[25] Sawera M, Gorodkin J, Cirera S, Fredholm M (2005). Mapping and expression studies of the mir17-92 cluster on pig chromosome 11.. Mamm Genome.

[26] Kim HJ, Cui XS, Kim EJ, Kim WJ, Kim NH (2006). New porcine microRNA genes found by homology search.. Genome.

[27] Kim J, Cho IS, Hong JS, Choi YK, Kim H (2008). Identification and characterization of new microRNAs from pig.. Mamm Genome.

[28] Huang TH, Zhu MJ, Li XY, Zhao SH (2008). Discovery of porcine microRNAs and profiling from skeletal muscle tissues during development.. PLoS ONE.

[29] McDaneld TG, Smith TP, Doumit ME, Miles JR, Coutinho LL (2009). MicroRNA transcriptome profiles during swine skeletal muscle development.. BMC Genomics.

[30] Xie SS, Huang TH, Shen Y, Li XY, Zhang XX Identification and characterization of microRNAs from porcine skeletal muscle.. Anim Genet.

[31] Cho IS, Kim J, Seo HY, Lim DH, Hong JS Cloning and characterization of microRNAs from porcine skeletal muscle and adipose tissue.. Mol Biol Rep.

[32] Reddy AM, Zheng Y, Jagadeeswaran G, Macmil SL, Graham WB (2009). Cloning, characterization and expression analysis of porcine microRNAs.. BMC Genomics.

[33] Nielsen M, Hansen JH, Hedegaard J, Nielsen RO, Panitz F MicroRNA identity and abundance in porcine skeletal muscles determined by deep sequencing.. Anim Genet.

[34] Sharbati S, Friedlander MR, Sharbati J, Hoeke L, Chen W Deciphering the porcine intestinal microRNA transcriptome.. BMC Genomics.

[35] Altschul SF, Gish W, Miller W, Myers EW, Lipman DJ (1990). Basic local alignment search tool.. J Mol Biol.

[36] Glazov EA, Cottee PA, Barris WC, Moore RJ, Dalrymple BP (2008). A microRNA catalog of the developing chicken embryo identified by a deep sequencing approach.. Genome Res.

[37] Friedlander MR, Chen W, Adamidi C, Maaskola J, Einspanier R (2008). Discovering microRNAs from deep sequencing data using miRDeep.. Nat Biotechnol.

[38] Zuker M (2003). Mfold web server for nucleic acid folding and hybridization prediction.. Nucleic Acids Res.

[39] Chen C, Ridzon DA, Broomer AJ, Zhou Z, Lee DH (2005). Real-time quantification of microRNAs by stem-loop RT-PCR.. Nucleic Acids Res.

[40] Li M, Xia Y, Gu Y, Zhang K, Lang Q (2010). MicroRNAome of Porcine Pre- and Postnatal Development.. PLoS ONE.

[41] Fernandez-Valverde SL, Taft RJ, Mattick JS (2010). Dynamic isomiR regulation in Drosophila development.. RNA.

[42] Chiang HR, Schoenfeld LW, Ruby JG, Auyeung VC, Spies N (2010). Mammalian microRNAs: experimental evaluation of novel and previously annotated genes.. Genes Dev.

[43] Kutay H, Bai S, Datta J, Motiwala T, Pogribny I, Frankel W, Jacob ST, Ghoshal K (2006). Downregulation of miR-122 in the rodent and human hepatocellular carcinomas.. J Cell Biochem.

[44] Esau C, Davis S, Murray SF, Yu XX, Pandey SK, Pear M (2006). miR-122 regulation of lipid metabolism revealed by in vivo antisense targeting.. Cell Metab.

[45] Wang Y, Lu Y, Toh ST, Sung WK, Tan P, Chow P, Chung AY, Jooi LL, Lee CG (2001). Lethal-7 is down-regulated by the hepatitis B virus x protein and targets signal transducer and activator of transcription 3.. J Hepatol.

[46] Tzur G, Israel A, Levy A, Benjamin H, Meiri E, Shufaro Y (2009). Comprehensive gene and microRNA expression profiling reveals a role for microRNAs in human liver development.. PLoS One.

[47] Tsang WP, Kwok TT (2010). Epigallocatechin gallate up-regulation of miR-16 and induction of apoptosis in human cancer cells.. J Nutr Biochem.

[48] Watanabe T, Takeda A, Mise K, Okuno T, Suzuki T (2005). Stage-specific expression of microRNAs during Xenopus development.. FEBS Lett.

[49] Thomson JM, Parker J, Perou CM, Hammond SM (2004). A custom microarray platform for analysis of microRNA gene expression.. Nat Methods.

[50] Smyth GK (2004). Linear models and empirical Bayes methods for assessing diferential expression in microarray experiments. Stat. Appl. Genet. Mol.. Biol.

