# Supplementary figures and images for: Discovery of Porcine microRNAs in Multiple Tissues by a Solexa Deep Sequencing Approach
Source: PLoS One. 2011 Jan 25;6(1):e16235. doi: 10.1371/journal.pone.0016235 (PMC3026822; doi:10.1371/journal.pone.0016235)

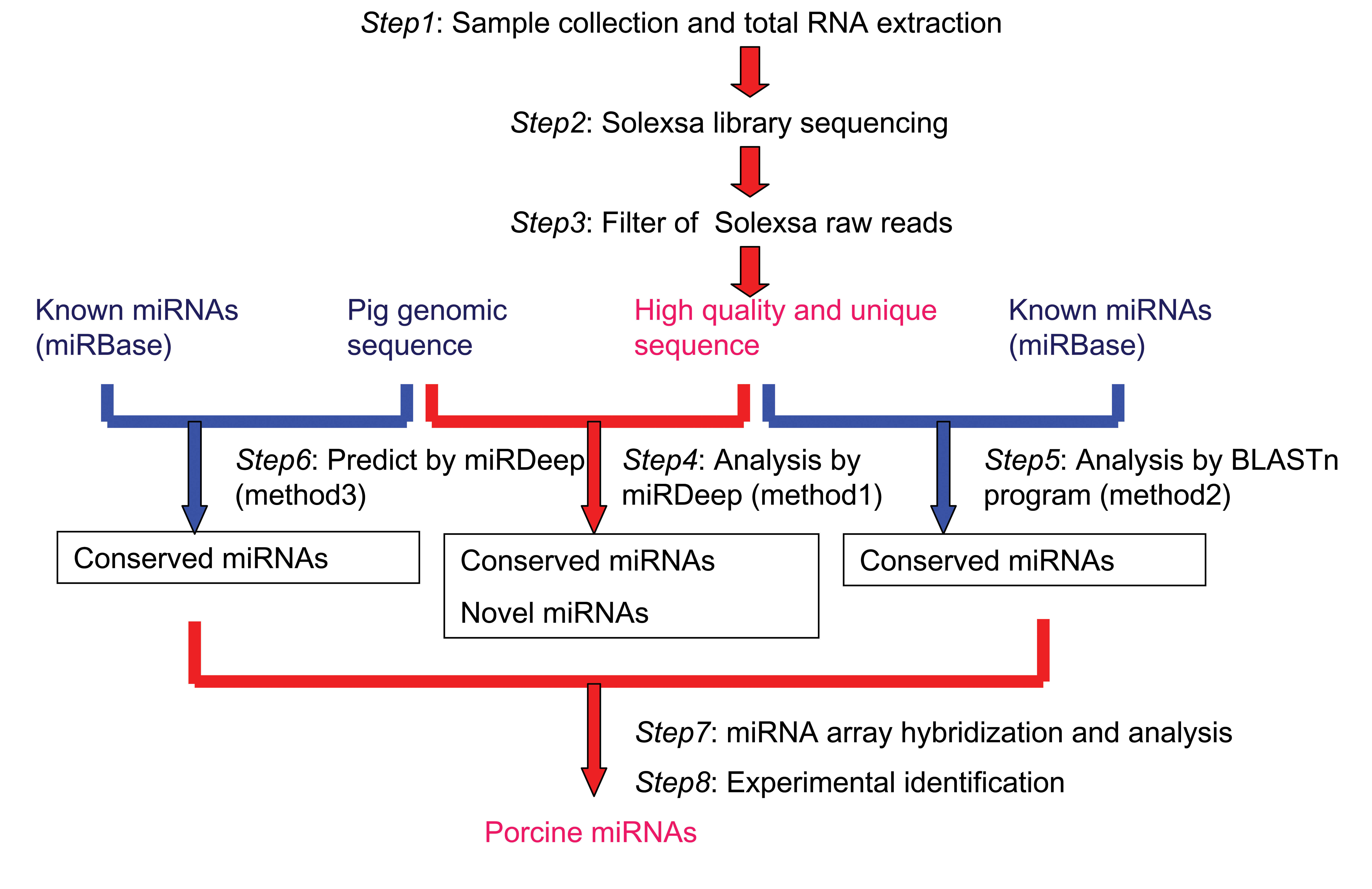

Supplement: Figure S1 — Illustration of high-throughput identification of porcine microRNAs by deep sequencing. (TIF) [file pone.0016235.s001.tif]
